# Botulinum toxin A versus microwave thermolysis for primary axillary hyperhidrosis: A randomized controlled trial

**DOI:** 10.1016/j.jdin.2023.12.011

**Published:** 2024-01-23

**Authors:** Gabriela Lladó Grove, Katrine Togsverd-Bo, Claus Zachariae, Merete Haedersdal

**Affiliations:** a Department of Dermatology, Copenhagen University Hospital – Bispebjerg, Copenhagen, Denmark; b Department of Dermatology, Copenhagen University Hospital – Gentofte, Copenhagen, Denmark; c Department of Clinical Medicine, University of Copenhagen, Copenhagen, Denmark

**Keywords:** botulinum toxin, bromhidrosis, hyperhidrosis, individualized treatment, microwave thermolysis, osmidrosis, personalized therapy, PROMs, QoL, randomized controlled trial, RCT

## Abstract

**Background:**

Botulinum toxin A (BTX) and microwave thermolysis (MWT) represent 2 treatment modalities for axillary hyperhidrosis with different procedural and efficacy profiles.

**Objective:**

To compare long-term outcomes following BTX vs MWT treatment of axillary hyperhidrosis.

**Methods:**

A prospective, randomized, within-patient, controlled trial, treating axillary hyperhidrosis with contralateral BTX and MWT. Objective sweat measurement and patient-reported outcome measures for sweat and odor were collected at baseline, 6-month and 1-year follow-up (6M/1YFU). Hair reduction and patient treatment preference was also assessed.

**Results:**

Sweat reduction was significant (all *P* <.01) for both interventions throughout the study. Objectively, sweat reduction was equal at 1-year FU (Δ*P* =.4282), but greater for BTX than MWT at 6-month FU (Δ*P* =.0053). Subjective sweat assessment presented comparable efficacy (6MFU: Δ*P* =.4142, 1YFU: Δ*P* =.1025). Odor reduction was significant (all *P* <.01) following both interventions, whereas only sustaining for MWT (6MFU: Δ*P* =.6826, 1YFU: Δ*P* =.0098). Long-term, hair reduction was visible after MWT, but not BTX (Δ*P* ≤.0001), and MWT was preferred by the majority of patients (76%).

**Limitations:**

The intrinsic challenges in efficacy assessment.

**Conclusion:**

This study exhibited BTX and MWT with similar sweat reduction, but distinguishable odor and hair reduction at 1-year FU. These findings support individualized treatment approaches for axillary hyperhidrosis based on patient-specific symptoms and preferences.

**Capsule Summary**

• Botulinum toxin A and microwave thermolysis for axillary hyperhidrosis have been investigated individually, whereas this side-by-side trial provides comparable evidence of their advantages and limitations.
• The clinical profiles of botulinum toxin A and microwave thermolysis differ significantly and should induce an individualized treatment approach depending on patient-specific symptoms and preferences.

## Introduction

Primary axillary hyperhidrosis is present in ≥2% of the general population^1^ and is known to significantly reduce quality of life (QoL) in affected patients due to the extreme sweating and often accompanying malodor.^2^ Disease severity is generally based on the objective gravimetric test,^3^ but patient-reported outcome measures (PROMs) are gaining more ground as they can assess the impact of both sweat, odor, and QoL in a standardized way.

The condition is managed through a wide variety of treatments, including topicals, systemic treatments and procedures as injectables, energy-based devices, and surgery.^4^ Different treatment options allow increasingly tailored treatments with focus on patient-specific symptoms and preferences.

Botulinum toxin A (BTX) has gained its place as standard treatment for focal hyperhidrosis in the past decades. Intradermal toxin injections induce blockage of acetylcholine release and hence the neuronal signal to sweat glands.^5^ Treatment efficacy has generally proven to be high, but the effect is temporary, most studies reporting average efficacy of 6 to 9 months, but varying between ≤4 months and ≥12 months.^6^, ^7^, ^8^, ^9^, ^10^

Microwave thermolysis (MWT) is a newer, energy-based treatment that irreversibly targets the sweat glands by delivering thermal energy to the dermal-fat interface.^11^^,^^12^ The device offers an alternative efficacy profile for axillary hyperhidrosis as well as osmidrosis and hair reduction^13^, ^14^, ^15^ that may be permanent.

Multiple studies have previously been presented on BTX,^5^, ^6^, ^7^, ^8^, ^9^, ^10^ individually, and MWT-focused literature has been expanding the past years.^11^, ^12^, ^13^, ^14^, ^15^, ^16^ However, in the light of the increasing demand for individualized treatment approaches, there is a lack of direct comparison of BTX and MWT in terms of clinical profile, efficacy and longevity, especially.

This study aimed to do a long-term side-by-side comparison of BTX and MWT for axillary hyperhidrosis, with both objective and subjective outcome measures, for the elucidation of the treatments’ individual procedural and efficacy profile, highlighting similarities and distinctions.

## Materials and Methods

### Design

This study was a prospective, randomized, within-patient, controlled trial of axillary hyperhidrosis treatment with BTX vs MWT at baseline followed by a telephone survey on day 2 and clinical follow-up (FU) up to 6-months and 1-year posttreatment.

The GCP-monitored trial was carried out between 3rd quartile 2021 and 2nd quartile 2023 at the Department of Dermatology, Copenhagen University Hospital, Denmark, and conducted in accordance with the Helsinki Declaration. Approvals from the Danish Medicines Agency (EudraCT: 2021–000877–10), the Regional Health Research Ethics Committee in Copenhagen (H–21013548) and the Danish Data Protection Agency (P-2021-436) were secured. Registration on clinicaltrials.gov (NCT05057117, ID: MWT-BTXA) was completed before study start. Informed consent was obtained from study patients prior to inclusion.

### Population

Adults with confirmed primary axillary hyperhidrosis at baseline, with a positive gravimetric test (women: ≥50 mg/5 minutes, men: ≥100 mg/5 minutes) and subjective rating of 3 or 4 on the Hyperhidrosis Disease Severity Scale (HDSS), were included.

Exclusion criteria comprised presence of generalized hyperhidrosis, medicine known to affect sweat secretion, pregnancy or lactation, previous axillary surgery, other medical condition in axillae, contraindications for BTX or MWT, history of intolerance to utilized medicinal products, and other hyperhidrosis treatment prior to inclusion defined as follows: topical prescription medication (≤10 days), systemic treatment (≤10 days), BTX (≤12 months), and MWT (ever).

Simultaneous therapy was not allowed, except for over-the-counter antiperspirants up until 1 to 5 days (type-dependent) before study visits.

### Interventions

All baseline treatments were performed by a single, trained clinician (GLG), treating one axilla with BTX and the contralateral with MWT.

Computerized randomization, equally distributing treatments between right/left axilla in blocks of 6, was performed by an independent data analyst and then kept in sealed, opaque envelopes until revealed in the given baseline session.

Treatment areas were assessed individually and defined by the anatomic features, including the visual full hair-bearing area, and supported by the starch-iodine test.

BTX treatment was executed with the commercially available Botox (Allergan Pharmaceuticals plc) with a standard 50 to 100 units (in 3-6 mL isotone sodium-chloride solution) per axilla depending on individual site size.

The miraDry-system (miraDry Inc) was utilized for the MWT treatment with standard setting of energy level 5 (5.8 GHz, 3.0 seconds) and standardized tumescent anesthesia of 70 to 124 mL (lidocaine-adrenaline [50 mL; 10 mg/mL + 5 μg/mL] in sodium-chloride solution) per axilla based on size and individual anesthetic effect.

### Patient characteristics

Demographics included age, sex, body mass index, and history of treatment with study interventions. Outcome measures were individually assessed for each axilla at baseline, 6-month and 1-year FU. Additionally, possible adverse effects were surveyed throughout the study duration.

### Objective outcome measures

#### Gravimetric test

Efficacy assessment for sweat was conducted with gravimetric testing using standardized filter paper (Whatman Ashless Quantitative Filter Paper, 9.0 cm diameter, grade 40). For all the tests, patients were in resting position in normal room temperature and had avoided antiperspirants ≥24 hours before testing. Weighing the filter paper before and 5 minutes after placement, at least one axilla produced ≥50 mg/5 minutes for women and ≥100 mg/5 minutes for men for a positive gravimetric test result, and the contralateral axilla at minimum 20% below the sex-specific threshold.

#### Starch-iodine test

Clinical visualization was collected with a digital camera, both with and without the starch-iodine test, which appears as purple-black coloration by application of iodine and corn starch in contact with sweat. The test supported the clinical assessment of treatment area.

#### Hair reduction

Axillary hair reduction following BTX and MWT until 1-year FU was each assessed by the clinician, supported by clinical images, on a 3-point scale (0-3: 0: none, 1: light, 2: moderate, and 3: severe) compared with baseline.

### Subjective outcome measures

#### PROMs

**Fig 1:**
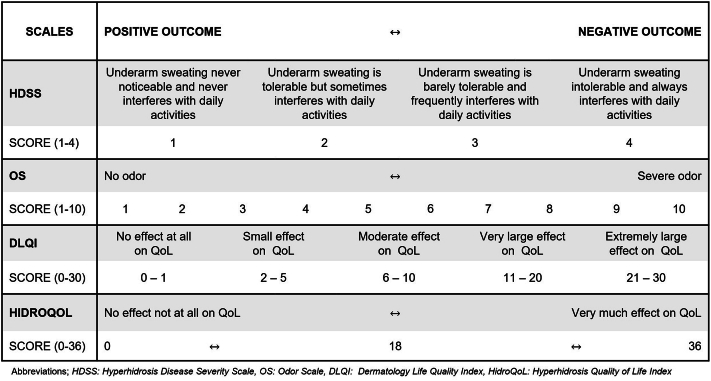
Patient-reported outcome measures assessment scales.

PROMs were collected for the assessment of subjective efficacy and included the HDSS, a 4-point scale for sweat, Odor Scale, a 10-point scale for malodor, and Dermatology Life Quality Index (DLQI), a 10-item questionnaire (max score 30), as well as Hyperhidrosis Quality of Life Index (HidroQoL), an 18-item questionnaire (max score 36). In all scales, high scores represented negative outcome and low scores positive outcome for the patient. See PROM scales overview in Fig 1.

#### Pain

Procedural pain during the treatment session was assessed for BTX and MWT on a standard 10-point Numerical Rating Scale. Postprocedural pain following BTX and MWT, was each assessed on a 3-point scale (0-3: 0: none, 1: light, 2: moderate, and 3: severe) on day 2 (telephone survey), and at clinical FU visits.

#### Patient satisfaction and preference

At the end of study, patient satisfaction and preferred treatment was registered. The overall treatment satisfaction was assessed on a 3-statement Likert scale (“Satisfied,” “Indecisive,” and “Not satisfied”), whereas patients’ preferred treatment entailed the whole 1-year experience with BTX vs MWT (“BTX preference,” “MWT preference,” and “Indecisive”).

### Statistics

Sample size calculation was based on the HDSS scale (1-4) with the expected, clinically meaningful change to be ≥1.0. Using calculations for a 2-tailed test of hypothesis (α = 0.05, power = 80%, SD = 1.2) the sample size was deemed to be 23 subjects. Considering a possible 25% drop out, 30 subjects were deemed sufficient for study inclusion.

Patient characteristics were presented with descriptive statistics, accordingly. Outcome measures were reported with absolute values for categorical measures and for continuous measures also by percentual change. No outcomes measures were normally distributed, so nonparametric test of paired data was applied (Wilcoxon signed-rank test) with medians and IQR.

*P-*values of <.05 were considered statistically significant; “*P*” values representing comparison of baseline and FU, whereas “Δ*P*” values representing difference between the 2 interventions (BTX and MWT). STATA v.14.2 (StataCorp LP) and R v.4.1.2 was used.

## Results

### Patient characteristics

A total of 30 patients with primary axillary hyperhidrosis were included and completed the study. Two-thirds were women and one-third were men with overall median age of 26 years. Prior to study participation, no patients had received MWT treatment (per protocol), whereas 8 (27%) had previously received BTX-treatments. See Table I for demographic overview.

**Table I:** Baseline demographics and clinical characteristics

Characteristics	Population
Women, *n* (%)	20 (67)
Age, y, median (IQR)	26 (23-35)
BMI, kg/m^2^, median (IQR)	23.1 (20.5-25.2)

Previous treatment	Subpopulation
Microwave thermolysis	0 (0)
Botulinum toxin A, *n* (%)∗	8 (27)
Botulinum toxin A, no. of treatments, median (IQR)	4 (2.5-5)

*N* = 30 patients.

*BMI*, Body mass index.

∗ ≥12 months before baseline.

All patients received a single baseline treatment with BTX in one axilla and MWT in the contralateral, according to the randomization. There was no significant downtime and no unexpected side effects following either intervention throughout the study, and no serious adverse events were registered.

### Objective outcome measures

#### Gravimetric test

Baseline gravimetric tests were bilaterally positive (medians 151 and 155 mg/5 minutes) with no significant difference between the 2 sides (Δ*P* =.6656). Sweat reduction was significant throughout the study following both BTX and MWT (*P* <.0001 for both). At 1-year FU, treatment efficacy was similar (median %-reduction BTX: 79% and MWT: 73% [Δ*P* =.4282]), whereas BTX performed better than MWT at 6-month FU (median %-reduction BTX: 74% and MWT: 58% [ΔP =.0053]). See Table II for detailed overview of gravimetric test results.

**Table II:** Objective measure of sweat intensity by gravimetric test at baseline, 6-months, and 1-year following botulinum toxin A and microwave thermolysis, presented with medians (IQR) and %-decrease

Outcome assessment	Gravimetric test		Δ*P* value
	BTX	MWT	BTX vs MWT
Baseline			
BL median (IQR), mg/5 min	151 (106-235)	155 (91-263)	.6656
6-month follow-up			
6-month follow-up median (IQR), mg/5 min	32 (20-89)∗	66 (35-95)∗	.0124∗∗
% median reduction (IQR) from BL	74 (56-86) %	58 (35-77) %	.0053∗∗
1-year follow-up			
1-year follow-up median (IQR), mg/5 min	35 (19-76)∗	47 (24-80)∗	.9508
% median reduction (IQR) from BL	79 (50-88) %	73 (34-87) %	.4282

*N* = 30 patients.

*%*, Percentual; *BL*, baseline; *BTX*, botulinum toxin A; *MWT*, microwave thermolysis.

*∗P* value BL vs FU: .0000. ∗∗Δ*P* value baseline versus follow-up: .0000. value BTX vs MWT: <.05.

#### Starch-iodine test

**Fig 2:**
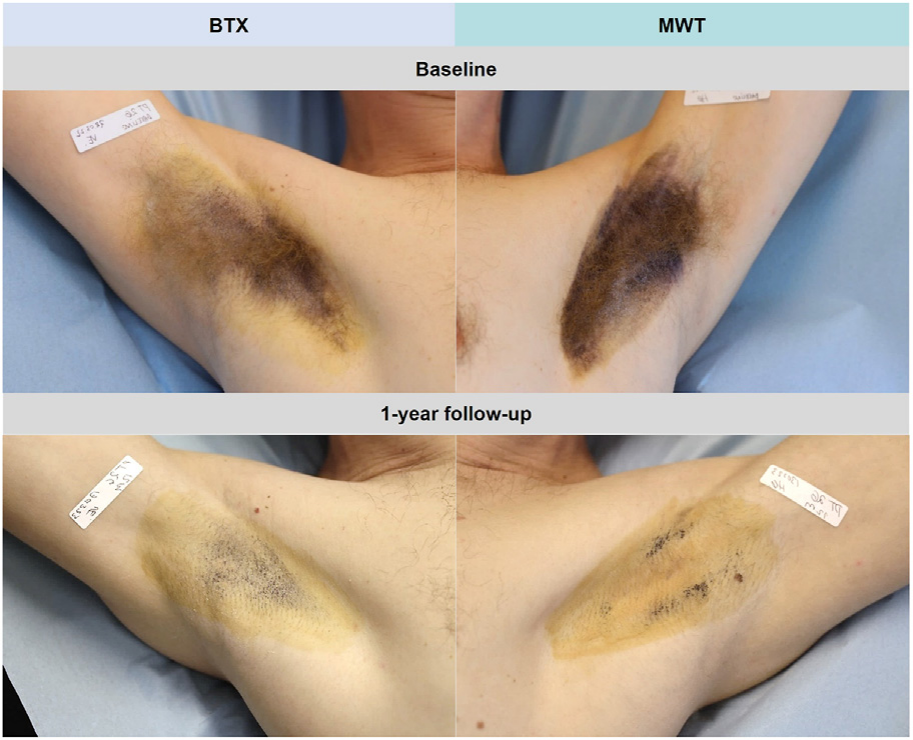
Sweat intensity visualized by starch-iodine test at baseline and 1-year following botulinum toxin A and microwave thermolysis in 1 study patient.

Supporting the objective findings, starch-iodine test was performed at baseline and FU for an instantaneous visualization of axillary sweat. As exemplified in Fig 2*,* similar sweat at baseline was visibly reduced bilaterally at 1-year FU with characteristic patterns for BTX and MWT, respectively.

#### Hair reduction

**Fig 3:**
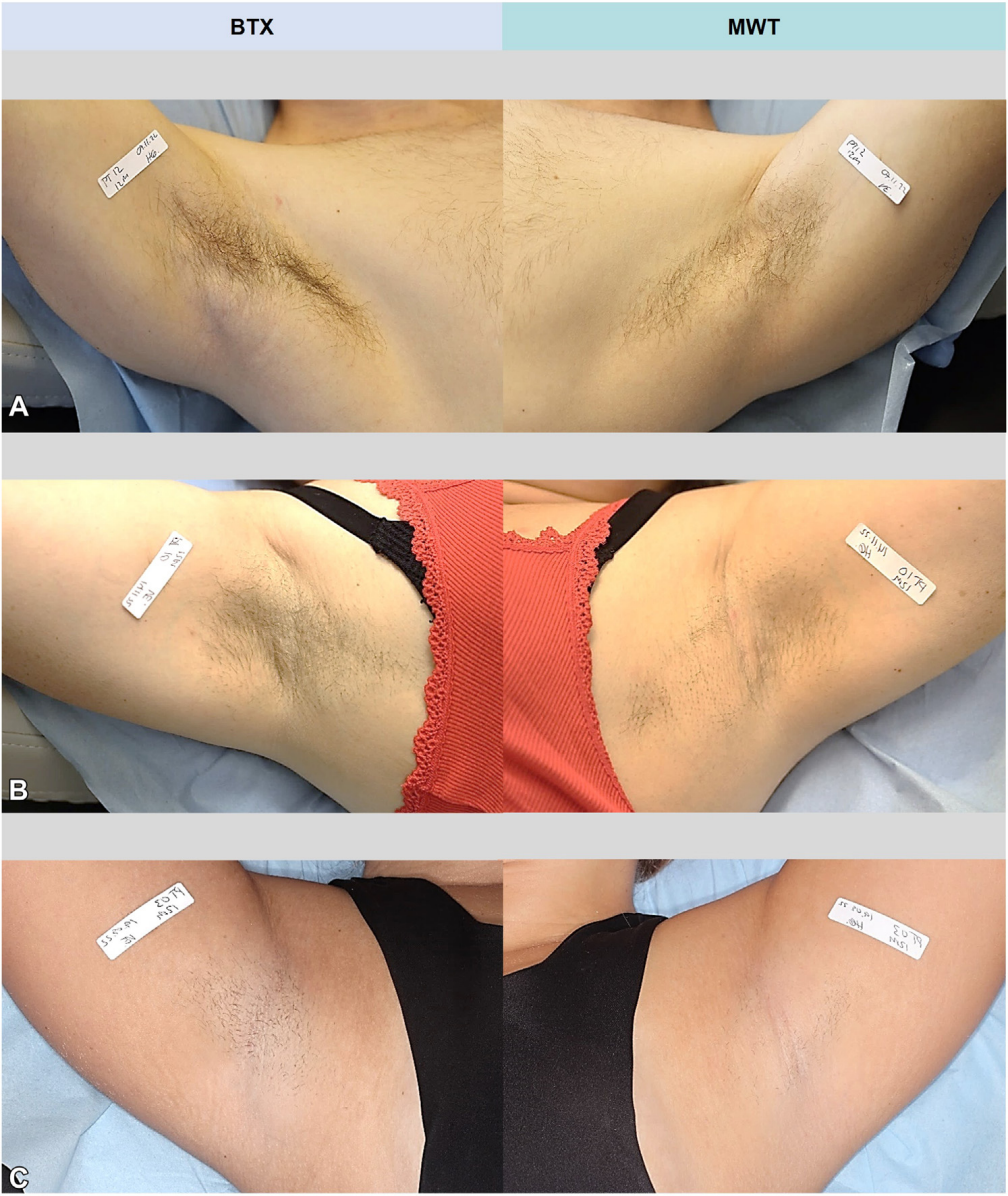
Axillary hair growth 1-year following botulinum toxin A and microwave thermolysis (MWT); examples of visible hair reduction in 3 study patients with **(A)** light, **(B)** moderate, and **(C)** severe hair reduction on the MWT-treated axilla.

Patients had no hair reduction following BTX treatment (median 0, IQR: 0-0), whereas visible moderate-severe hair reduction (median 2, IQR: 2-3) was significant on the MWT-treated side (Δ*P* ≤.0001) at 1-year FU. See Fig 3 for examples of visible 1-year FU hair reduction.

### Subjective outcome measures

#### Sweat

At baseline patients reported severe hyperhidrosis bilaterally (HDSS medians 3), which both treatments significantly reduced throughout the study (HDSS medians 2, *P* <.0001 for both BTX and MWT at 6MFU and 1YFU). Subjective treatment efficacy was similar between BTX and MWT at either time point (6MFU: Δ*P* =.4142, 1YFU: Δ*P* =.1025).

#### Odor

Patient-reported odor was moderate-severe at baseline on both sides (Odor Scale medians 6.5). Odor reduction was significant following both treatments, but at 1-year FU it started to revert for the BTX-treated side (6MFU: median 3 [*P* =.0018], 1YFU: median 4 [*P* =.0106]), whereas the odor reduction sustained on the MWT-side (6MFU: median 2.5 [*P* =.0002], 1YFU: median 2.5 [*P* =.0001]). Efficacy on odor was similar at 6-month FU, but distinguishable on the long-term (6MFU: Δ*P* =.6826, 1YFU: Δ*P* =.0098).

#### QoL

Both scales identified moderate-severe affection on QoL at baseline (DLQI: medians 10, HidroQoL: medians 22). For either treatment, QoL improvement was significant throughout the study on both DLQI (6MFU: medians 3, 1YFU: medians 4 [*P* ≤.0001 for all]) and HidroQoL (6MFU: medians 8.5, 1YFU: median 11.5 [BTX] and 11 [MWT] [*P* ≤.0001 for all]). Overall, both scales showed a similar effect on QoL from treatment with BTX and MWT (DLQI; 6MFU: Δ*P* =.0857, 1YFU: Δ*P* =.3745, HidroQoL; 6MFU: Δ*P* =.4374, 1YFU: Δ*P* =.1761).

**Fig 4:**
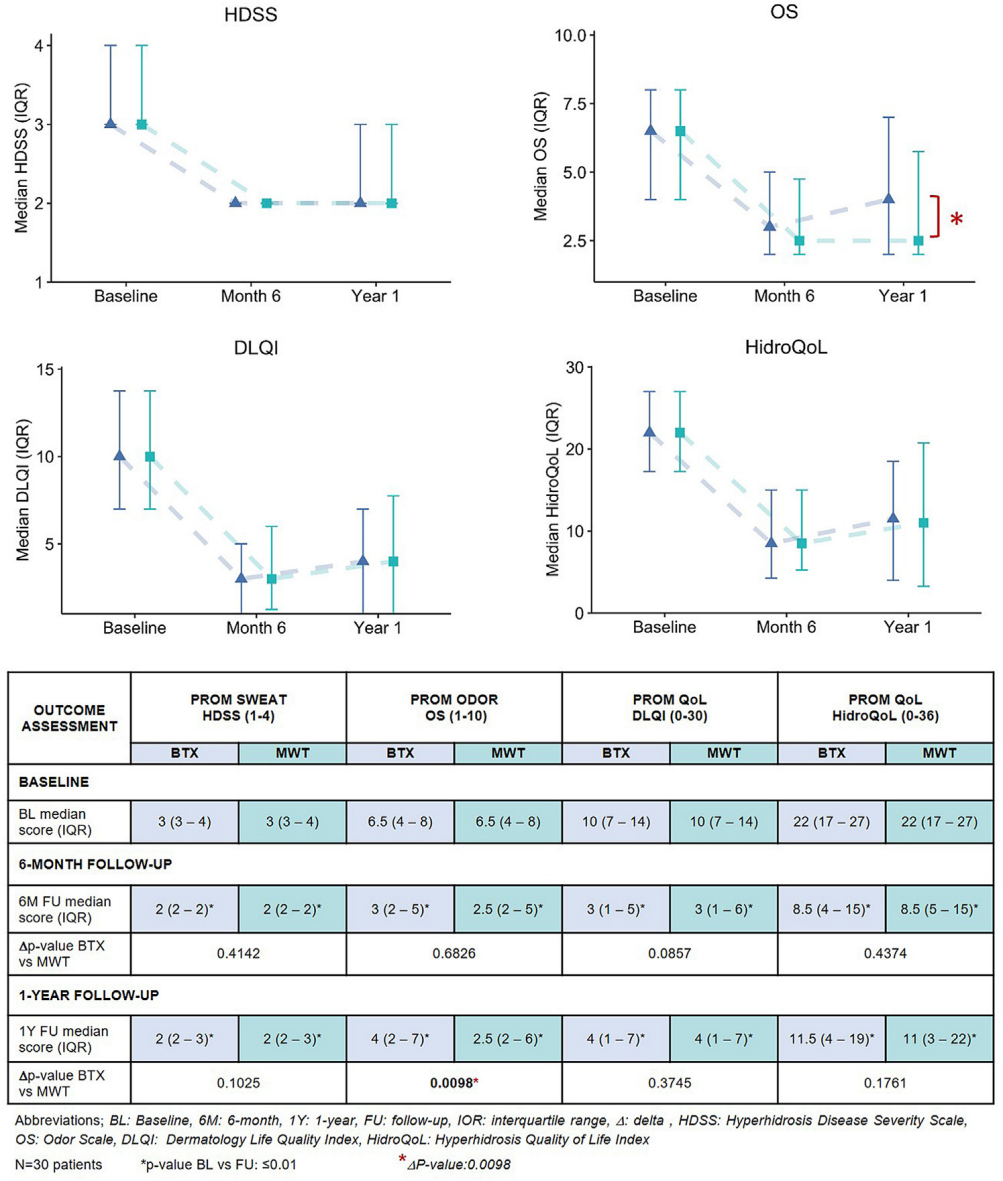
Subjective measures of sweat, odor, and quality of life at baseline, 6-months and 1-year following botulinum toxin A and microwave thermolysis, presented with medians (IQR) and Δ*P* values.

See Fig 4 for detailed and graphical overview of outcome measures.

#### Pain

During treatments, patients assessed the procedural pain on-site. Procedural pain was higher for BTX (median 4.5, IQR: 3-7) than for MWT (median 3, IQR: 2-4) (Δ*P* <.0001).

On the telephone survey day 2, however, postprocedural pain was reported by the patients as light-moderate for MWT (median 1, IQR: 1-2), whereas there was none for BTX (median 0, IQR: 0-0) (Δ*P* =.3173).

#### Patient satisfaction and preference

Patients were asked to provide overall treatment satisfaction; 24 (80%) were satisfied, 2 (7%) were indecisive, and 4 (13%) were not satisfied due to insufficient effect on both sweat and odor.

At end of study, patients were asked about their treatment preference for their continued individual approach, when taking all into account; procedure, side effects and efficacy. MWT was preferred by 23 (76%), BTX preferred by 5 (17%), while 2 (7%) were indecisive. There was no sex-specific preference difference.

## Discussion

This study conducted a side-by-side comparison of BTX and MWT for axillary hyperhidrosis in a randomized, controlled trial with 1-year FU. Both treatments effectively improved axillary sweat, odor as well as QoL. While BTX performed objectively better than MWT for sweat reduction at 6-month FU, they performed similarly on sweat reduction at 1-year FU. In contrast, odor reduction at 6-months was similar, but at 1-year FU, odor reduction started to revert on the BTX-side, while it sustained on the MWT-side. On-site procedural pain was higher for BTX, whereas postprocedural pain was only reported for MWT. Furthermore, hair reduction was the sole long-term side effect, only seen after MWT.

The patients in this study showed a distinctive preference for MWT at the end of study. Most patients reported that acceptable efficacy with MWT, combined with the possibility to avoid continuous treatment, was preferred. Some patients preferred the short-term efficacy of BTX, whereas others did not experience satisfactory effect of either treatments, but mainly pursued a second MWT treatment, poststudy. Eight patients had previous BTX treatment, but the majority were naïve to both treatments before inclusion.

### Strengths and limitations

The randomized, controlled trial design was the main strength of this comparative investigation, but blinding was not feasible. Follow-up time up to 1 year was long, but even longer FU, allowing BTX to wear off completely, could have added to the study findings. Patients were included exclusively on hyperhidrosis parameters, and as such the difference in odor reduction may have been bigger with osmidrosis as an additional inclusion criteria.

The standardized collection of both objective and subjective measures provided broader and more credible outcome assessment than with either measure, individually. Efficacy assessment is complicated, however, by the nature of this condition, existing within a spectrum from normal to pathologic sweating, varying significantly over the course of a day. Despite the intraindividual design, the intrinsic challenges with the objective gravimetric test is a limitation, and coherently, PROMs also underlie risk of bias.^17^ Although PROMs scales have proven to be useful in this patient group,^16^^,^^18^, ^19^, ^20^, ^21^, ^22^ the patients’ ability to distinguish between sides is dependent on the respective scale properties. For the QoL scales, specifically, it did not appear that patients were able to significantly distinguish between the 2 sides, despite noticeable differences in both sweat and odor over the course of the study.

### Perspectives

A progressive demand and focus on individualized treatment pillars clinicians’ effort toward better outcomes and increased patient satisfaction. Patients consider both efficacy and side effects, especially long-term. While pain will be the main concern for some, others will predominantly be impatient for immediate and/or long-term efficacy. A prior study on MWT confirmed that side effects are generally temporary and that patients are able to accept these in exchange of satisfactory efficacy.^16^ Because of the different clinical efficacy profiles, some patients may prefer and/or clearly benefit more from one treatment than the other. Additional outcomes, such as hair reduction, can even be desirable to some patients, whereas others may have a specific aversion to this.

Although rare, serious adverse events have been reported both to BTX and MWT. It remains important to take these into account for optimal, individualized treatment plans.

Long-term use of BTX may increase the risk of toxic effects which appears to be highly dose dependent, and adverse events are reported more common with therapeutic BTX use than cosmetic use.^23^ Toxin spread from injections sites may cause serious adverse that can present as muscle weakness, severe dysphagia or respiratory compromise, but necrotizing fasciitis has also been reported after BTX in alternative treatment areas.^24^, ^25^, ^26^ For hyperhidrosis, specifically, BTX has been described to cause botulism-like generalized weakness.^27^ Fatal outcomes have been related to local or systemic toxin spread causing respiratory arrest, gangrene or anaphylaxis.^24^^,^^28^^,^^29^ Notably, patients with neuromuscular diseases are more vulnerable to BTX-related adverse events.^26^

Treatment with microwave devices can cause potentially serious adverse events, and infections are the most common, while burns and neurologic complications have also been reported.^30^ A case with fatal necrotizing fasciitis following nonstandardized MWT of alternative treatment areas has been described.^31^ For standard MWT in the axillae, specifically, mainly neuropathy has been described in relation to brachial plexus affection, particularly in thin individuals and in cases without hydro-dissection with tumescent anesthesia.^32^, ^33^, ^34^, ^35^ Current standard MWT treatment includes extensive tumescent anesthesia for reducing the risk of neuropathy.^36^ Furthermore, studies have shown negative MWT-related outcomes in patients with concomitant hidradenitis suppurativa.^37^, ^38^, ^39^

The risk of serious side effects cements the importance of these treatments being provided by health care professionals that can take medical history, including drug allergies and contraindications, into account as well as assure relevant prevention and handling of possible serious complications.

Optimizing the patient experience is the key to increased patient satisfaction, whether it be based on efficacy, alone, or the possible side effects as well as additional outcomes. The clinical profile of each tailored treatment option, the patient needs and medical history, as well as the patient expectations should be considered.

## Conclusion

This study adds evidence to treatment similarities and distinctions with a direct comparison of BTX and MWT up to 1-year FU. Treatment with BTX vs MWT present with very different clinical profiles and the patients’ focus and preference in this study were the long-term outcomes. The findings are relevant for future patient information, useful for patient-involvement in shared decision-making, and support the increased demand for individualized treatments based on patient-specific symptoms and preferences.

## Conflicts of interest

Dr Haedersdal has received speaker honorarium for microwave thermolysis symposium. Drs Grove, Togsverd-Bo, and Zachariae have no conflicts of interest to declare.
